# Exocrine Pancreatic Function in Girls with Anorexia Nervosa

**DOI:** 10.3390/nu13093280

**Published:** 2021-09-20

**Authors:** Żaneta Malczyk, Wojciech Roczniak, Bogdan Mazur, Jarosław Kwiecień, Katarzyna Ziora, Karolina Górska-Flak, Joanna Oświęcimska

**Affiliations:** 1 Chair and Department of Pediatrics, Faculty of Medical Sciences in Zabrze, Medical University of Silesia in Katowice, ul. 3 Maja 13/15, 41-800 Zabrze, Poland; jkwiecien@sum.edu.pl (J.K.); ziorkasia@wp.pl (K.Z.); 2 Institute of Medicine, Jan Grodek State University in Sanok, ul. Mickiewicza 21, 38-500 Sanok, Poland; wojciech_roczniak@interia.pl (W.R.); smina@poczta.onet.pl (J.O.); 3 Department of Microbiology and Immunology, Faculty of Medical Sciences in Zabrze, Medical University of Silesia in Katowice, ul. Jordana 19, 41-808 Zabrze, Poland; bmazur@sum.edu.pl; 4 Department of Pediatrics, Institute of Medicine, University of Opole, Al. Wincentego Witosa 26, 45-401 Opole, Poland; karolagf2204@gmail.com

**Keywords:** anorexia nervosa, breath test, exocrine pancreatic function, ghrelin, leptin

## Abstract

Objectives: To assess pancreatic exocrine function in patients with anorexia nervosa using a breath test with ^13^C-labeled mixed triglycerides (MTG-BT) and to determine the relationship between the test results and selected biochemical and hormonal parameters. Material and methods: Anthropometric measurements, biochemical and hormonal parameters (serum leptin, soluble leptin receptor (sLR), acylated and desacylated ghrelin, free leptin index (FLI)), and MTG-BT were performed in a group of 31 girls with the restrictive type of AN, as well as 38 healthy girls (C). Results: The average cumulative dose of ^13^C-triglycerides recovered with exhaled air (%CD) was similar in both study groups, while the average time from ^13^C-triglycerides administration to peak ^13^CO_2_ excretion in expired air (time to peak (TTP)) was significantly longer in patients with AN compared to C. In both groups, %CD correlated negatively with FLI. TTP correlated negatively with sLR and FLI in the AN and with serum insulin and HOMA-IR values in the C. Conclusions: In girls with AN, the pancreatic efficiency of lipase secretion was found to be normal, while the kinetics of this enzyme secretion were disturbed. These changes may result from disorders in the functioning of the adipose–insular and islet–acinar axes.

## 1. Introduction

Anorexia nervosa (AN) is a chronic disease syndrome, the essence of which is deliberate weight reduction by using both quantitative and qualitative diet restrictions and other activities such as increased physical activity, provoked vomiting, the use of laxatives and diuretics, and body cooling [1]. This severe psychiatric disorder is characterized by a high incidence of coexisting psychiatric conditions, treatment resistance, and a substantial risk of death from medical complications and suicide [2]. The diagnostic criteria of AN are presented in the Diagnostic and Statistical Manual of Mental Disorders of the American Psychiatric Association, fifth edition [3]. The two designated subtypes of AN are the restricting subtype, which is characterized by dietary restriction, and the binge-eating and purging subtype, in which restriction is accompanied by binge eating, purging, or both; the condition may progress from one subtype to another [2].

In the United States, the prevalence of the AN is estimated to be 0.3–2% [4]. The male-to-female ratio is approximately 10:1 to 15:1 [5]. AN is the third most common chronic illness among adolescents, after obesity and asthma [6].

The etiology of AN is multifactorial and includes biological (genetic determinants and neurobiochemical disorders), psychological, environmental, and social factors [7]. The risk factors for developing the disease include female gender, adolescence, obesity, family history (eating disorders, depression, and obsessive–compulsive disorders), abnormal relationships with parents, obsessive–compulsive personality, prematurity, and low birth weight [8]. There is good evidence that genetic factors play an important role in the pathogenesis of AN. Based on studies of monozygotic twins, it was found that their influence ranges from 28% to 58% [9]. Moreover, recently, a genomewide association study identified several risk loci for AN that were also predictive of other psychiatric disorders, as well as a low BMI and metabolic derangements [2]. The neuroendocrine changes associated with AN include disorders of serotonergic, dopaminergic, and opioid neurotransmission and abnormal secretion of appetite-stimulating hormones such as ghrelin and orexin [10].

As a result of starvation, functional disorders of virtually all organs and systems occur [1]. Patients with AN often report gastrointestinal symptoms such as postprandial fullness, epigastric pain, nausea, and flatulence [11]. These ailments usually intensify during realimentation, so that it can be assumed that at least some of them results from digestive disorders caused by impaired pancreatic exocrine function [1]. The literature data confirm that malnutrition may lead to impaired pancreatic exocrine function, although the pathomechanism of this phenomenon has not yet been fully explained [12,13].

Studies on pancreatic exocrine function in AN remain scarce [14,15,16,17,18,19,20]. We know these patients typically have an isolated increase in serum amylase activity or an increase in both amylase and lipase activity [14,18,19,20].

During treatment, particularly in patients with severe AN, it is advisable to monitor the functioning of their digestive tract. Due to the poor somatic and mental state of patients, it is important that the tests not burden them; therefore, we decided to use a non-invasive breath test with ^13^C-labeled mixed triglycerides (MTG-BT) to assess the exocrine function of the pancreas. In the available literature, only one report was found on the use of this test in patients with AN [17].

AN is associated with adaptive changes in multiple endocrine axes to help maintain euglycemia and energy homeostasis. Patients with AN have higher growth hormone and cortisol levels, lower levels of gonadotropins and gonadal hormones, triiodothyronine, insulin, and oxytocin [21]. Moreover, hormone secretion disorders (e.g., leptin, ghrelin, and insulin) that can affect the exocrine function of the pancreas occur [22,23].

There are two forms of circulating leptin—active free leptin and inactive—associated with receptors [24]. The main circulating blood leptin binding protein is the OB-Re receptor [25]. Due to the phenomenon of leptin resistance, total serum concentrations of this hormone may not reflect its biological activity. For this reason, it is recommended to calculate the so-called free leptin index (FLI), which is the ratio of total leptin concentration to soluble leptin receptor (OB-Re) concentration [26]. It is well known that in people with normal body weight, as well as in obesity, leptin levels are proportional to the body fat content and positively correlate with BMI [27,28]. Patients with anorexia nervosa have significantly reduced serum leptin levels and free leptin index values, while soluble leptin receptor levels are significantly higher compared to those with normal body weight [27,28,29]. The presence of leptin receptors on pancreatic acinar cells suggests that this hormone may participate in the direct regulation of pancreatic enzyme secretion [23,30,31].

The pancreatic exocrine function is closely related to its endocrine function in a morphological and functional way. Insulin is the best-known element of the islet–acinar axis. Insulin is thought to affect the secretion of pancreatic enzymes in a paracrine manner by directly binding to its receptors on follicular cells [32]. Reduced insulin levels and HOMA-IR (homeostatic model assessment insulin resistance) values are observed in patients suffering from anorexia nervosa [33].

The ghrelin concentration is currently considered to be an important indicator of the energy state. Starving and lowering the glucose level raise the concentration of this hormone and activates orexin neurons [34]. Numerous publications confirm that elevated ghrelin levels are observed in patients suffering from anorexia nervosa, and its secretion does not undergo postprandial inhibition as in healthy people [35,36,37,38,39,40]. There are two active molecular forms of ghrelin—acylated and desacylated [41,42]. The acylated ghrelin molecule stimulates a group of AgRP/NPY neurons in the hypothalamus, increasing the secretion of orexygenic substances stimulating appetite [41,42]. It also stimulates hydrochloric acid and gastrin release and gastric peristalsis [42]. Desacylated ghrelin accounts for approximately 80% of total ghrelin in plasma. In contrast to the acylated form, it causes a negative energy balance and reduces body weight by reducing food intake and delaying gastric emptying in mice [42]. The effect of ghrelin on pancreatic exocrine activity has not been clearly determined. It is presumed that it can take place by vasovagal reflexes or modulation of CCK secretion, as well as its effect on insulin secretion [23,43].

Because the changes in the concentrations of the aforementioned hormones typically found in AN may affect the pancreatic exocrine function, the authors of this study decided to analyze the relationship between the concentrations of these hormones, selected anthropometric and biochemical parameters, and MTG-BT results in a group of girls with AN and a healthy group.

## 2. Materials and Methods

This study was approved by the local ethics committee at the Medical University of Silesia (approval no. KNW/0022/KB1/95/15). All patients and their parents/representatives provided their informed consent prior to their inclusion in the study. The study was conducted on 69 girls aged 12–17 years. Table 1 presents the clinical characteristics of the groups studied (Table 1). The study group (AN) consisted of 31 girls aged 12–17 years with restriction type of AN diagnosed according to the DSM-5 criteria [3]. The exclusion criteria were purging type of AN, known chronic concomitant disease, other mental illnesses diagnosed by a psychiatrist, abnormal results of additional laboratory tests (serum electrolytes, aspartate and alanine aminotransferases, creatinine, IgA concentration, and antitissue transglutaminase IgA antibodies titer), taking medications including hormonal drugs and/or dietary supplements within the past three months, and infections within the last month before the study. All patients were hospitalized and the tests were performed during the first three days after admission, before starting treatment.

The control group (K) consisted of 38 healthy girls aged 13–17 with normal body weight and BMI according to age and gender norms in the Polish population [44]. The exclusion criteria were known chronic concomitant disease, abnormal results of additional laboratory tests (serum electrolytes, aspartate and alanine aminotransferases and creatinine), taking medications including hormonal drugs and/or dietary supplements within the past 3 months, infections within the last month before the study.

All examined girls were Tanner IV-V. Two girls with anorexia nervosa had primary amenorrhea, the remaining 29 had amenorrhea secundaria lasting on average 5.4 ± 2.4 months (1.0–10.0 months). All control girls were eumenorrheic.

Anthropometric measurements (weight, height, and BMI), basic biochemical parameters (total cholesterol, HDL, LDL, triglycerides, glucose, bilirubin, alanine (ALT) and asparagine aminotransferase (AST), gamma-glutamyltranspeptidase (GGTP), and total amylase), and hormonal parameters (insulin, leptin, soluble leptin receptor (sLR), acylated, and desacylated ghrelin) were assessed in both examined groups. The HOMA-IR insulin resistance ratio was calculated based on fasting glucose and insulin concentrations [45]. The free leptin index (FLI) was calculated as the ratio of total leptin concentration to soluble leptin receptor concentration [26].

A ^13^C-mixed triglyceride (MTG-BT) breathing test was performed in the examined groups. The test was performed after fasting, after an 8 h night break in eating meals. After the methodology was explained to the patient, the first sample of exhaust air was collected (zero sample), asking the subject to take a deep breath, hold the air for 5 s, and then slowly breathe out into the coated aluminum bag through a disposable mouthpiece with a non-return valve (Wagner Analysen Technik GmbH, Bremen, Germany). After the exhaust air sample was obtained, the bag was sealed with a cork. The subject then received a test breakfast, consisting of 50 g of white bread, on which was spread chocolate nut cream with the addition of 150 mg of ^13^C-mixed triglyceride, which the patient was to eat within 10 min, drinking 200 mL of still mineral water. The amount of chocolate nut cream (containing 32% fat) was 0.64 g/kg (0.2 g of fat/kg).

After the test breakfast was eaten, further samples of exhaled air were taken at 30 min intervals for 6 h in a similar way to a zero sample. During the breath test, the girls did not take food or liquids. They remained sitting or lying down.

Exhaled air analysis was performed on an IRIS apparatus from Wagner GmbH (Bremen, Germany). The content of ^13^CO_2_ relative to ^12^CO_2_ was determined in all samples. The increase in ^13^CO_2_ concentration from baseline was determined as DOB (delta over baseline), according to the formula:

DOB [‰] = δPDB_x_ [‰] − δPDB_0_ [‰]

where:

x—sample collected after x minutes;

0—zero sample;

δPDB (*Pee Dee Belemnite*)—unit determining the share of ^13^CO_2_ in the total CO_2_ pool in relation to the international ^13^C/^12^C isotope ratio pattern, which is limestone from the Cretaceous formation (belemnite fossil) in Pee Dee, SC, USA.

Based on the DOB results obtained, two parameters were calculated using the IRIS software to assess ^13^C triglyceride metabolism:

- Cumulative dose of ^13^C-triglycerides recovered from exhaled air, defined as the percentage of ^13^CO_2_ excreted relative to the amount of ^13^C-triglycerides administered (% of cumulative dose (%CD);
- Time from administration of ^13^C-triglycerides to obtaining the peak ^13^CO excretion (time to peak (TTP)).

The obtained results were compared to the values obtained in the control group, as well as to the standards given by the apparatus manufacturer, according to which the cut-off threshold of %CD indicating insufficient pancreatic lipase secretion is <22%.

### Statistical Analysis

The database was prepared using Excel 2000 (Microsoft Corporation). Statistical analysis was carried out using Statistica 10.0 software (StatSoft Inc., Tulsa, OK, USA). A normal data distribution was assessed using the Kolmogorov–Smirnov test; the homogeneity of variance was computed using Levene’s test. We employed intergroup comparisons using the Student’s *t*-test or the Mann–Whitney *U*-test if the distribution of variables differed significantly from normal and if the Levene’s test showed no homogeneity of variance. To compare the number of subjects who obtained incorrect MTG-BT results with the number of subjects whose test results were normal, the *χ*^2^ test was used. The effect size for significant differences was established using Cohen’s d coefficient. The level of the linear relationship between random variables was established by determining Pearson’s coefficients in the case of variables with normal distribution or Spearman’s rank correlation coefficient if the distribution of variables significantly deviated from normal. *p*-values < 0.05 were considered statistically significant.

## 3. Results

Results of the biochemical and hormonal parameter assessment are shown in Table 2.

**Figure 1:**
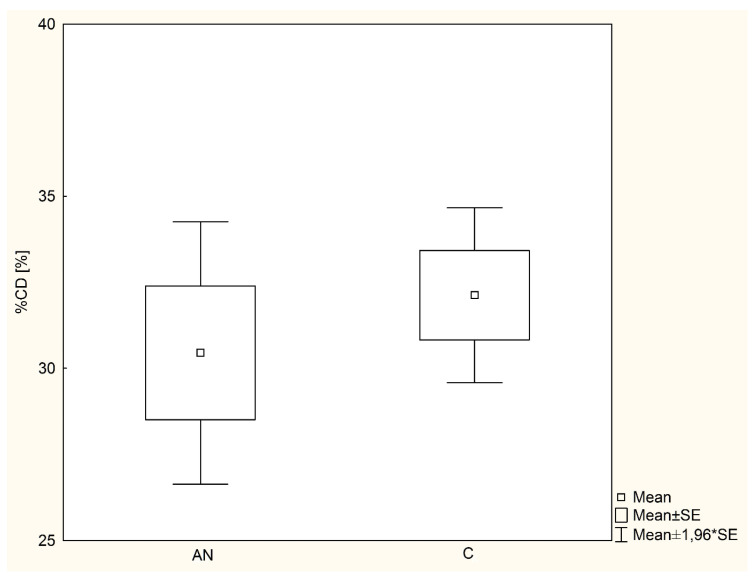
Cumulative dose of ^13^C-triglycerides recovered with expiratory air in MTG-BT in examined girls with anorexia nervosa and healthy controls. *p* = 0.49; SE—standard error; AN—anorexia nervosa; C—healthy controls; %CD—cumulative dose of ^13^C-triglycerides recovered with expiratory air in MTG-BT.

The average cumulative dose of ^13^C-triglycerides recovered with expiratory air (%CD) in the AN group (30.4 ± 10.8%) was similar to that of the healthy girls (32.1 ± 7.9%) (Figure 1).

The values of %CD indicating pancreatic exocrine failure in the lipase range (<22%) were found in six girls with anorexia nervosa (7.8%) and two subjects from the control group (2.6%). There was no statistically significant difference in the frequency of abnormal %CD values between the study groups (*χ*^2^ test, *p* = 0.11).

**Figure 2:**
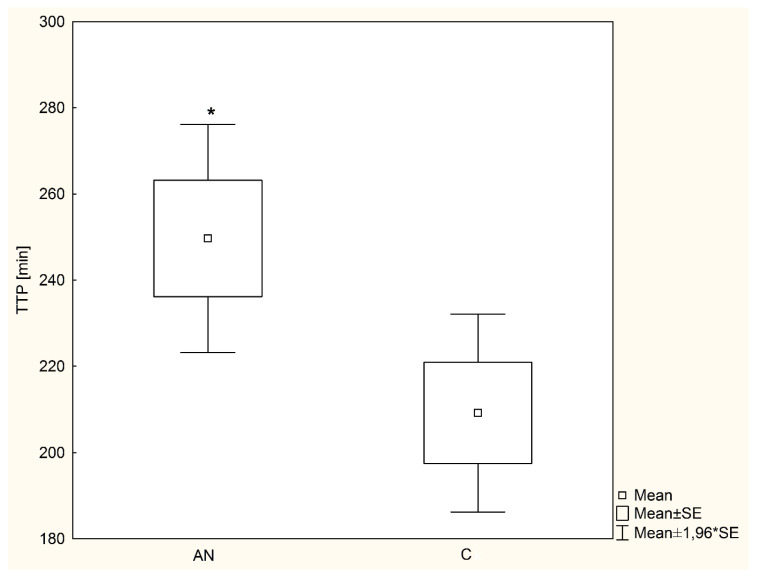
The average time from administration of ^13^C-triglycerides to achieving peak ^13^CO_2_ excretions in the exhaled air (time to peak (TTP)) in examined girls with anorexia nervosa and healthy controls. * *p* = 0.03; Cohen’s d = 0.54; SE—standard error; AN—anorexia nervosa; C—healthy controls; TTP—time to peak.

The average time from administration of ^13^C-triglycerides to achieving peak ^13^CO_2_ excretions in the exhaled air (time to peak (TTP)) in the AN group (249.6 ± 75.3 min) was statistically significantly (*p* = 0.03) longer compared to the group control (209.0 ± 71.2 min) (Figure 2).

There were no statistically significant correlations between the anthropometric parameters and the results of the body mass composition analysis, as well as the results of biochemical parameter determinations and %CD and TTP in both examined groups.

A strong negative correlation between %CD and FLI was noted in both groups. Significant negative correlations between TTP, sLR concentration, and FLI values were found in the group of patients with AN. In the control group, statistically significant negative correlations were found between %CD and desacylated ghrelin concentration, and between TTP and insulin concentrations and HOMA-IR values (Table 3).

## 4. Discussion

Severe malnutrition may significantly impair pancreatic enzyme secretion, and the pathogenesis of this phenomenon is still widely discussed [46]. In malnourished monkeys, chronic energy deficiency has been shown to cause structural changes in the pancreas [46]. These observations have also been confirmed in humans, in whom malnutrition may lead to atrophy of pancreatic alveolar cells and increased zymogen release [46,47].

There are only a few studies on pancreatic exocrine function in anorexia nervosa, and their results are often divergent scarce [14,15,16,17,18,19,20].

In the course of AN, an isolated increase in serum and urine amylase activity is most often observed, especially in the purging type, which is an effect of stimulation of the parotid gland [14]. There are also reports in the literature on increased amylase activity, also in the form of restrictive AN, but the pathomechanism of this phenomenon has not been fully explained. Cox et al. [19] found elevated serum amylase activity in seven of the 10 patients with AN that they examined. However, they did not show its relationship with the ultrasound examination of the pancreas and clinical symptoms and the results of biochemical tests normalized after successful re-feeding. Several studies have evaluated both serum amylase and lipase activity. However, their elevated activities were not associated with any clinical symptoms or abnormalities of the ultrasound image of the pancreas and were normalized after weight gain [14,15,20].

In our study, we showed that the mean time from the administration of ^13^C-triglycerides in MTG-BT to the peak of ^13^CO_2_ excretion in the exhaled air in the AN group was statistically significantly longer than in the control group, while the values of the cumulative ^13^C-triglyceride dose recovered with expiratory air did not differ from those obtained in healthy girls.

Thus far, only one publication has been published, in which Martinez et al. [17], similarly to us, assessed the exocrine function of the pancreas in patients with AN based on a non-invasive ^13^C-labeled mixed triglyceride breath test and fecal elastase activity assay. Additionally, a D-xylose test was performed to rule out malabsorption. The study was conducted in 10 adult women with anorexia nervosa, aged 26 ± 1.19 years. Seven subjects suffered from the restrictive form of the disease, and three from the purging form. The assays were performed twice—in the acute phase of the disease during hospitalization and after the target BMI of ≥20 kg/m^2^ was reached. The cited authors considered the %CD >29% to be the normal value. In nine patients, no functional disorders of the pancreas or malabsorption disorders were found, and in one patient with abnormal test results, a diagnosis of celiac disease coexisting with AN was finally established [17]. The %CD results that we obtained did not differ between the studied groups, which is consistent with the observations of Martinez et al. [17]. Unfortunately, these authors did not specify TTP.

Because pancreatic lipase is the main factor responsible for the digestion of fat, and gastric lipase hydrolyzes only 15% of lipids, the obtained results may suggest that the total amount of this enzyme secreted after the eating of a test meal by patients with anorexia nervosa is similar to that of the control group, while in patients with AN, the peak of its secretion is delayed [48]. Therefore, it can be assumed that girls with anorexia nervosa reveal only disorders of the kinetics of pancreatic enzyme secretion, and not pancreatic insufficiency.

One of the reasons for the observed TTP delay in girls with AN may be the delayed gastric emptying, which prolongs the transit time of the ^13^C-labeled triglycerides to the distal gastrointestinal tract. Admittedly, Maes et al. [49] found no significant relationship between the degree of its severity and the rate of gastric emptying in patients with pancreatic insufficiency. On the contrary, in people without pancreatic dysfunction, the degree of reduction in lipolysis was correlated with the time of gastric emptying. This is the situation we may observe in patients with anorexia nervosa; moreover, previous studies have confirmed that the rate of gastric emptying in patients with AN is much lower than that in healthy subjects [50]. According to some authors, this phenomenon occurs only after the consumption of solid foods [51], while others have shown that delayed gastric emptying applies to both solid and liquid foods [50]. Thus far, it has not been established whether gastric motility disorders in anorexia nervosa are temporary, associated with thinness, or permanent [50,51].

### 4.1. Leptin

The presence of leptin receptors on pancreatic acinar cells suggests that this hormone may participate in the direct regulation of pancreatic enzyme secretion [23,30,31,52]. However, the literature data on the effect of leptin on the exocrine function of the pancreas are sparse and predominantly include in vitro and animal studies.

Elinson et al. [31] found, in an in vitro study on pancreatic cells of the AR42J line, that leptin directly inhibits both intracellular expression and pancreatic lipase secretion. This observation was confirmed in an experiment carried out by Jaworek et al. [23], who showed that intraperitoneal administration of leptin to rats does not affect the basal secretion of pancreatic enzymes, but inhibits their postprandial secretion in a dose-dependent manner. An interesting report on the influence of leptin on the exocrine pancreatic function was published by Matyjek et al. [53]. They observed that the intravenous injection of leptin in rats at a dose of 1 μg/kg bw. inhibits the pancreatic secretion of protein and amylase only after the previous administration of CCK-8. A ten-times higher dose of intravenous leptin continued to inhibit the secretion of pancreatic juice, protein, and amylase, while their enteral administration had the opposite effect. The increase in pancreatic exocrine activity after enteral administration of this hormone was closely related to the increase in serum concentrations of CCK-8, and the blockade of CCK-1 receptors, as well as the blockade of the vagus nerve endings in the duodenal and small intestine muscular membranes and vagotomy, completely abolished the effect of enteral administration of leptin. A high dose of this hormone administered intravenously also inhibited the exocrine pancreatic activity induced by the intravenous injection of 2-deoxyglucose [23,53]. The results of the above studies suggest that the mechanism of action of leptin on exocrine pancreatic activity is complex and may be direct or indirect through modulation of the activity of the intestinal and central nervous system, as well as through influencing the secretion of enterohormones [30].

The current state of knowledge does not allow for an unequivocal explanation of the negative correlations between %CD and FLI in both studied groups. It should be emphasized, however, that in girls with anorexia nervosa, no reduction in the %CD value was found, and the results obtained in the majority of the studied patients allowed for the exclusion of exocrine pancreatic insufficiency. It can only be speculated that the observed correlations may have resulted from the central action of leptin, which increases the sympathetic nervous system activity and inhibits the parasympathetic activity [54]. It cannot be excluded that the mechanisms regulating the secretion of pancreatic lipase in girls with anorexia nervosa and healthy girls are different and, in the latter, may include disorders of the neurotransmitter systems, in particular the central serotonergic system [23] or disorders of the secretion of CCK and other enterohormones [55].

In our study, we observed that in girls with AN, the maximum lipase secretion occurred later than in the control group, and the TTP values were negatively correlated with the FLI values and soluble leptin receptor. In the available literature, we did not find any reports on the influence of leptin on the kinetics of lipase secretion. Perhaps in patients with anorexia nervosa, the shift in peak secretion of this enzyme over time is associated with delayed gastric emptying. Kentish et al. [56] proved that leptin has a two-way action on nerve endings in the gastric mucosa, depending on the nutritional status. Namely, after long-term restriction of food consumption, its stimulating effect on the mechanoreceptors in the gastric mucosa, which transmits information about the degree of its filling via the vagus nerve, and the inhibitory effect of leptin on the nerve endings in the muscular membrane of this organ, stimulated by larger pieces of food, come to the fore, which can lead to abnormal gastric kinetics. It cannot be excluded that the observed TTP delay in patients with anorexia nervosa is one of the elements of the global disturbance of the gastrointestinal kinetics in this disease [11].

### 4.2. Insulin

The exocrine function of the pancreas is closely related to its endocrine function in a morphological and functional manner. Direct evidence of the existence of such a relationship is the fact that the destruction of pancreatic islets with streptozocin results in atrophy of this organ and its exocrine dysfunction [57]. The best-known component of the islet– acinar axis is insulin. Animal studies have shown that insulin has a direct effect on the basal and stimulated secretion of pancreatic amylase, while reports of its effect on pancreatic exocrine function in humans are sparse and contradictory, probably due to imperfect research techniques [58]. Insulin is believed to affect the secretion of pancreatic enzymes in a paracrine manner by directly binding to its receptors on follicular cells [57].

Indirect evidence of the existence of the islet–acinar axis has been provided by studies of the exo- and endocrine pancreatic function in diabetic patients. Many studies have confirmed the effect of insulin on the secretion of pancreatic enzymes in patients with types 1 and 2 of this disease, whose progression leads to morphological changes in the pancreas in the form of steatosis and fibrosis, reduction of organ mass, and disorders of its exocrine function [57]. Pathological changes in the exocrine pancreas may also lead to disturbances in its endocrine function and glucose metabolism. Diseases of this organ, such as inflammation or cancer, usually cause hyperglycemia and the so-called pancreatic diabetes [59].

The negative correlations that the authors found between insulin concentrations and the values of the insulin resistance coefficients HOMA-IR and TTP confirm the modulating effect of insulin on the exocrine pancreatic activity in healthy girls and are consistent with the above-discussed reports. Interestingly, such relationships have not been demonstrated in patients with AN, which indicates a different nature of the regulation of the exocrine pancreatic function in this disease. Insulin concentrations and HOMA-IR values are very low in anorexia nervosa, which is consistent with literature reports [37]. Perhaps, at low concentrations in fasting states, insulin does not have a significant effect on the kinetics of pancreatic lipase secretion, and the regulation of its secretion is different than in healthy subjects. It cannot be excluded that such a relationship exists but is not linear. The authors measured only fasting insulin levels, but the exocrine activity of the pancreas in MTG-BT may also be influenced by changes in serum insulin levels after administration of the test meal, which, apart from fat, also contained carbohydrates, especially because the two groups significantly differ in their sensitivity to insulin. This issue requires clarification in the course of further research.

### 4.3. Ghrelin

The influence of ghrelin on the exocrine function of the pancreas has not been clearly established. Perhaps this results from the different biological effects of its acylated and desacylated forms. Although the expression of GHS-R ghrelin receptors on pancreatic cells of the AR42J line was found, this hormone did not affect the secretion of amylase by isolated sections of this organ in vitro [23]. Zhang et al. [60] showed that the intravenous administration of ghrelin to rats reduces the secretion of pancreatic enzymes stimulated by CCK acting on intra-pancreatic neurons. In turn, injection of this hormone into the central nervous system stimulates the exocrine activity of the pancreas via the vagus nerve. Ghrelin administered to the lumen of the duodenum acts similarly, and this effect depends on CCK and the activation of intra-pancreatic weight-vagal reflexes [23,57].

The effect of ghrelin on the exocrine pancreatic activity may also take place through its influence on insulin secretion, although the exact mechanism of this interaction has not been determined thus far, and experimental studies have produced conflicting results [57]. In humans, administration of large amounts of acylated ghrelin causes a rapid increase in glucose and insulin levels. In healthy subjects, a physiological increase in plasma ghrelin does not alter circulating levels of glucose, insulin, C-peptide, or glucagon. Administration of a high dose of desacylated ghrelin does not affect insulin secretion. It has been observed that when both forms of ghrelin are administered simultaneously, its desacylated form prevents an increase in glucose and insulin concentrations induced by the acylated form and significantly improves the insulin sensitivity of tissues [42].

In our study, the concentrations of acylated and desacylated ghrelin in patients with anorexia nervosa were higher than in the control group, which is consistent with the results published by other researchers [61]. However, the results of postprandial AN ghrelin levels are inconsistent. Physiologically, in healthy people, ghrelin levels decrease after a meal. However, in patients with AN, this phenomenon has not been observed or the decreases in acylated ghrelin concentrations caused by meals are moderate and delayed [61]. It cannot be ruled out that the biological effect of ghrelin in anorexia nervosa is weakened due to the development of resistance to this hormone [61]. In this study, only fasting acylated and desacylated ghrelin concentrations were determined; however, it can be assumed that changes in ghrelin concentrations and the proportion between the concentrations of its acylated and desacylated forms after the test meal may affect the exocrine activity of the pancreas, which requires more detailed studies.

In healthy girls, a negative correlation was found between the concentrations of desacylated ghrelin and TTP. Such a relationship was not found in the AN group. This may indicate that ghrelin does not affect the secretion of pancreatic lipase in this disease and that its secretion regulation is different than in healthy subjects.

### 4.4. Limitations of This Work and Directions for Further Research

The gastric emptying rate was not determined in this study. Because delayed gastric emptying in AN may affect the exocrine function of the pancreas, and in particular the kinetics of lipase secretion, it is necessary to conduct a study in which the rate of gastric emptying using a radioisotope test is performed simultaneously with MTG-BT. However, considering that the test meals applied to measure these two parameters are different, new methodological studies on the concomitant assessment of pancreatic exocrine function and gastric emptying are needed. It is also advisable to additionally assess the concentrations of the tested hormones—leptin, soluble leptin receptor, acylated and desacylated ghrelin, and insulin—during the entire study at individual MTG-BT time points. This will allow for a more accurate determination of the relationship between the dynamics of changes in their concentrations and the rate of ^13^C elimination with breathing air. A prospective study on the influence of body weight restoration on pancreatic exocrine function is also needed.

## 5. Conclusions

Based on the conducted studies, we conclude that in girls with anorexia nervosa, the efficiency of the pancreas in terms of lipase secretion is normal, but the kinetics of this enzyme secretion are disturbed. These changes are not related to the degree of malnutrition, but may result from disorders of the adipo–insular and islet–alveolar axes.

## Figures and Tables

**Table 1:** Clinical characteristics of the studied group of girls with anorexia nervosa (AN) and the healthy control (C).

Parameter	AN (*n* = 31)	C (*n* = 38)	*p* (Cohen’s d Coefficient)
	Mean ± SD (Min–Max)		
Age (years)	15.1 ± 1.6 (12.3–17.4)	15.9 ± 0.9 (13.4–17.7)	0.08
Weight (kg)	40.9 ± 6.4 (27.5–55.1)	55.4 ± 7.2 (40.6–73.0)	<0.0001 (−2.26)
Weight-SDS	−1.61 ± 0.82 (–3.36 to 0.45)	−0.10 ± 0.87 (–2.04 to 2.05)	<0.0001 (−1.84)
Height (m)	1.62 ± 0.78 (1.43–1.82)	1.63 ± 0.62 (1.49–1.74)	0.45
BMI (kg/m^2^)	15.38 ± 1.46 (10.7–17.58)	20.59 ± 2.06 (15.66–25.40)	<0.0001 (–3.56)
BMI-SDS	−1.82 ± 0.61 (−3.88 to −0.81)	−0.08 ± 0.76 (−1.85 to 1.77)	<0.0001 (−2.85)
Max weight before illness (kg)	58.0 ± 12.8 (32.00–90.00)	–	
Disease duration (months)	9.5 ± 7.1 (2.0–34.0)	–	
Loss of weight (kg)	17.1 ± 8.7 (4.5–37.2)	–	
Rate of loss of weight (kg/month)	2.3 ± 1.4 (0.4–6.9)	–	
Duration of amenorrhea (months)	5.4 ± 2.4 (1.0–10.0)	–	

AN—anorexia nervosa group; C—control group; SD—standard deviation; BMI—body mass index; SDS—standard deviation score.

**Table 2:** Results of the biochemical and hormonal parameter assessment in the examined group of girls with anorexia nervosa (AN) and the healthy control (C).

Parameter	AN (*n* = 31)	C (*n* = 38)	*p* (Cohen’s d Coefficient)
	Mean ± SD (Min–Max)		
ALT (U/L)	21.2 ± 18.8 (7.4–88.9)	12.4 ± 4.7 (5.0–28.1)	0.003 (0.46)
AST (U/L)	22.5 ± 13.8 (12.8–84.6)	18.0 ± 3.1 (13.8–28.2)	0.21
GGTP (U/L)	14.4 ± 12.4 (6.0–70.0)	12.3 ± 5.4 (6.0–35.0)	0.57
Total bilirubin (µmol/L)	10.4 ± 6.4 (2.5–27.0)	11.8 ± 11.2 (8.1–2.2)	0.53
Amylase (U/L)	54.5 ± 25.4 (22.0–149.0)	63.7 ± 21.8 (25.0–112.0)	0.11
Glucose (mg/dL)	79.75 ± 7.34 (53.3–91.0)	90.4 ± 8.7 (75.0–109.0)	<0.0001 (−1.45)
Insulin (µU/mL)	4.62 ± 2.49 (1.32–12.90)	10.82 ± 3.57 (4.61–18.62)	<0.0001 (−2.49)
HOMA-IR	0.92 ± 0.53 (0.25–2.68)	2.44 ± 088 (0.94–4.38)	<0.0001 (−2.87)
Leptin (ng/mL)	1.26 ± 1.57 (0.12–7.42)	11.51 ± 6.59 (1.16–25.28)	<0.0001 (−6.53)
sLR (ng/mL)	12.13 ± 5.62 (6.79–33.42)	6.27 ± 1.72 (3.57–13.32)	<0.0001 (1.04)
FLI	0.12 ± 0.18 (0.01 –0.92)	1.99 ± 1.35 (0.20–5.77)	<0.0001 (−10.39)
Acylated ghrelin (pg/mL)	168.24 ± 259.73 (22.53–1420.95)	52.01 ± 56.96 (18.93–289.92)	<0.0001 (0.45)
Desacylated ghrelin (pg/mL)	741.15 ± 387.46 (143.79–1813.08)	505.48 ± 178.74 (246.12–959.58)	<0.0001 (0.61)

AN—anorexia nervosa group; C—control group; SD—standard deviation; sLR—soluble leptin receptor; FLI—free leptin index.

**Table 3:** Analysis of the correlation between the results of hormonal tests and the cumulative dose of ^13^C-triglycerides recovered with exhaled air and the time from administration of ^13^C-triglycerides to achieving peak ^13^CO_2_ excretion.

Parameter	Group			
	AN (*n* = 31)		C (*n* = 38)	
	%CD (%)	TTP (min)	%CD (%)	TTP (min)
Insulin (µU/mL)	r = −0.18 *p* = 0.31	r = −0.11 *p* = 0.94	r = −0.11 *p* = 0.50	r = −0.36 *p* = 0.02
HOMA-IR	r = −0.16 *p* = 0.36	r = −0.01 *p* = 0.94	r = −0.07 *p* = 0.68	r = −0.36 *p* = 0.02
Leptin (ng/mL)	r = −0.26 *p* = 0.14	r = −0.13 *p* = 0.46	r = −0.14 *p* = 0.40	r = −0.21 *p* = 0.19
sLR (ng/mL)	r = 0.01 *p* = 0.99	r = −0.38 *p* = 0.03	r = 0.02 *p* = 0.87	r = −0.13 *p* = 0.42
FLI	r = −0.77 *p* < 0.0001	r = −0.36 *p* = 0.04	r = −0.61 *p* < 0.0001	r = −0.10 *p* = 0.55
Acylated ghrelin (pg/mL)	r = 0.23 *p* = 0.20	r = 0.15 *p* = 0.40	r = −0.07 *p* = 0.67	r = 0.25 *p* = 0.12
Desacylated ghrelin (pg/mL)	r = −0.02 *p* = 0.88	r = −0.23 *p* = 0.20	r = −0.41 *p* = 0.01	r = −0.01 *p* = 0.99

AN—anorexia nervosa; C—control group; %CD—cumulative dose of ^13^C-triglycerides recovered with expiratory air; TTP—time from administration of ^13^C-triglycerides to achieving peak ^13^CO_2_ excretion in the exhaled air; sLR—soluble leptin receptor; FLI—free leptin index.

## Data Availability

The data presented in this study are available on request from the corresponding author.
